# Phenotypes Associated with Second Chromosome *P* Element Insertions in *Drosophila melanogaster*

**DOI:** 10.1534/g3.116.030940

**Published:** 2016-06-10

**Authors:** Lily Kahsai, Gillian H. Millburn, Kevin R. Cook

**Affiliations:** * Bloomington *Drosophila* Stock Center, Department of Biology, Indiana University, Indiana 47405; † FlyBase, Department of Physiology, Development and Neuroscience, University of Cambridge, CB2 3DY Cambridge, United Kingdom

**Keywords:** *Drosophila melanogaster*, *P* element, insertional mutagenesis, complementation, phenotypic characterization

## Abstract

In *Drosophila melanogaster*, *P* element transposition has been a productive means of insertional mutagenesis. Thousands of genes have been tagged with natural and engineered *P* element constructs. Nevertheless, chromosomes carrying *P* element insertions tend to have high levels of background mutations from *P* elements inserting and excising during transposition. Consequently, the phenotypes seen when *P* element-bearing chromosomes are homozygous are often not attributable to the P insertions themselves. In this study, 178 strains in the Bloomington *Drosophila* Stock Center collection with P insertions on the second chromosome were complementation tested against molecularly defined chromosomal deletions and previously characterized single-gene mutations to determine if recessive lethality or sterility is associated with the P insertions rather than background mutations. This information should prove valuable to geneticists using these strains for experimental studies of gene function.

During *P* element transposition, it is common for a *P* element to insert into a chromosome and immediately excise, leaving a mutational footprint, before inserting into the genome in its final position (Cooley *et al.* 1988, Kania *et al.* 1995, Deak *et al.* 1997, Salzberg *et al.* 1997, Spradling *et al.* 1999, Ashburner *et al.* 2005). These “hit and run” events often disrupt genes; consequently, the phenotypes seen when insertion-bearing chromosomes are made homozygous cannot necessarily be attributed to the disruption of genes where *P* elements are located. Proof that a phenotype is associated with a *P* element insertion usually comes from reverting the phenotype upon transposase-mediated excision of the *P* element or showing that the P insertion fails to complement a loss-of-function mutation in the gene where the P insertion is found.

Most *P* element insertions from early *Drosophila* transposition screens were given symbols that reflected the phenotypes seen when the chromosomes were made homozygous (Spradling *et al.* 1999). For example, *P{lacW}l(2)k01209^k01209^* was named as a recessive lethal on the second chromosome [“*l(2)*”], while other insertions were named for phenotypes such as female sterility (*fs*) and male sterility (*ms*). The Berkeley *Drosophila* Genome Project put considerable effort into verifying the phenotypes of P insertions by complementation testing the insertions against chromosomal deletions, other P insertions, and previously characterized loss-of-function mutations (Spradling *et al.* 1999). In addition, many labs have investigated the phenotypes of particular insertions. Nevertheless, the purported phenotypes of hundreds of P insertions from early screens have never been confirmed, and insertions from more recent screens have generally been given phenotype-neutral symbols. In the study described here, we analyzed 178 second chromosome P insertions with potentially misleading symbols in the Bloomington *Drosophila* Stock Center collection to determine whether the insertions are responsible for recessive lethal or sterile phenotypes.

## Materials and Methods

Our approach was straightforward: we identified chromosomal deletions that encompass the insertion sites of *P* elements, made complementation crosses between deletion and P insertion stocks, and scored the appropriate progeny classes for lethality, female sterility, or male sterility. Assessing P insertions that had previously been localized to a specific genomic sequence often required only a single deletion cross. *P* elements that had previously been localized by *in situ* hybridization of polytene chromosome preparations were tested with a tiling series of deletions that spanned the genomic regions estimated to contain the insertions. Occasionally, single-gene mutations were complementation tested against the P insertions instead of deletions, or in addition to deletions. As positive controls, all deletion and single-gene mutation stocks were complementation tested against relevant, previously characterized, loss-of-function mutations (Supplemental Material, Table S1). All stocks were obtained from the Bloomington *Drosophila* Stock Center collection. All crosses were made on standard medium and reared under routine conditions. All genomic coordinates are given in terms of the Release 6 assembly. Polytene cytologies were estimated from Release 6 coordinates using FlyBase map conversion tables. Genetic symbols were changed to reflect our findings according to standard FlyBase nomenclatural practices.

### Data availability

Changes to FlyBase entries have been coordinated with this report and will appear in a 2016 FlyBase update. Strains may be obtained from the Bloomington *Drosophila* Stock Center.

## Results and Discussion

Table 1 shows the results of complementation tests with 95 *P* element stocks that indicate the recessive phenotypes seen when insertion chromosomes are made homozygous do not map to the P insertions themselves. Based on these results, we updated most of the insertion symbols. If the P insertion was positioned between genes, then the symbol was updated to show it as a simple insertion implying nothing about phenotypes. For example, *P{PZ}l(2)10333^10333^* was changed to *P{PZ}10333*. We updated 38 symbols in this way. If the *P* element insertion was positioned within the transcribed portion of an annotated gene, the symbol was updated to show the insertion as an allele of the gene, but the updated FlyBase entry indicates that no known phenotype is associated with the insertion. For example, *P{lacW}l(2)k09610^k09610^* was changed to *P{lacW}ush^k09610^*. We updated 47 symbols in this way. Since the lethality or sterility of these insertion chromosomes does not map to the insertion, and there is no way to know if it is the result of a single mutation or multiple mutations without further tests, the original gene and allele entries have been eliminated from FlyBase. For example, the *l(2)10333* and *l(2)k09610* gene entries, and the *l(2)10333^10333^* and *l(2)k09610^k09610^* allele entries have been eliminated. The gene and allele entries for noninsertion mutations were retained only when the phenotypes of the mutations have been characterized beyond simple lethality or sterility, or when the mutations are members of multiallele complementation groups. For example, *P{lacW}bdg^k08407^* now represents the site of a nonlethal P insertion, and *l(2)k08407^k08407^* represents the linked noninsertion lethal mutation formerly conflated under *P{lacW}l(2)k08407^k08407^*. In this case, it is necessary to have an allele entry for the noninsertion *l(2)k08407^k08407^* mutation because there is presumably a single mutation causing the imaginal disc abnormalities described in Roch *et al.* (1998) for the homozygous chromosome.

**Table 1:** Insertions with no associated lethal or sterile phenotype

Original Symbol	Previous Mapping*^a^*	Complementing Deletions and Mutations	Phenotype*^b^*	New Insertion Symbol	New Allele*^c^*
*P{f^+^13}l(2)37Co^1^*	*in situ*	*Df(2L)BSC341*	v	*P{f^+^13}37C*	
*P{lacW}rack^k15001^*	seq	*Df(2R)BSC160*, *Df(2R)ED2247*	v	*P{lacW}tou^k15001^*	
*P{hsneo}fs(2)neo9^1^*	*in situ*	*Df(2L)C144*	ff	*P{hsneo}neo9*	
*P{hsneo}fs(2)neo12^1^*	*in situ*	*Df(2R)ED2247*	ff	*P{hsneo}neo12*	
*P{Cp38*::*Adh}mfs(2)1^1^*	*in situ*	*Df(2L)ED1473*, *Df(2L)ED1378*	mf, ff	*P{Cp38*::*Adh}1*	
*P{lacW}l(2)k01301^k01301^*	seq	*Df(2R)FDD-0003576*	v	*P{lacW}CR43651^k01301^*	
*P{lacW}l(2)k01302^k01302^*	seq	*Df(2L)BSC693*	v	*P{lacW}tkv^k01302^*	*CR14033^k01302^*
*P{lacW}l(2)k02205^k02205^*	seq	*Df(2R)BSC482*	v	*P{lacW}sli^k02205^*	
*P{lacW}l(2)k02520^k02520^*	seq	*Df(2R)Exel6050*	v	*P{lacW}k02520*	
*P{lacW}l(2)k03110^k03110^*	seq	*Df(2R)ED3181*	v, h	*P{lacW}k03110*	
*P{lacW}l(2)k03111^k03111^*	seq	*Df(2R)BSC131*	v	*P{lacW}CG1648^k03111^*	
*P{lacW}l(2)k03204^k03204^*	seq	*Df(2R)BSC326*	v	*P{lacW}jing^k03204^*	
*P{lacW}l(2)k03609^k03609^*	seq	*Df(2R)Exel7142*	v	*P{lacW}k03609*	
*P{lacW}l(2)k05420^k05420^*	seq	*Df(2R)BSC131*	v	*P{lacW}k05420*	
*P{lacW}l(2)k05421^k05421^*	seq	*Df(2R)ED3181*	v, h	*P{lacW}CG6426^k05421^*	
*P{lacW}l(2)k05812^k05812^*	seq	*Df(2L)BSC145*	ff	*P{lacW}piwi^k05812^*	
*P{lacW}l(2)k06402^k06402^*	seq	*Df(2R)BSC154*	v	*P{lacW}k06402*	
*P{lacW}l(2)k06904^k06904^*	seq	*Df(2R)Exel7173*, *Df(2R)ED3952*	v	*P{lacW}k06904*	
*P{lacW}l(2)k07005^k07005^*	seq	*Df(2L)BSC107*	v	*P{lacW}k07005*	
*P{lacW}l(2)k07015^k07015^*	seq	*Df(2L)BSC855*	v	*P{lacW}k07015*	
*P{lacW}Sema-2b^k07127^*	seq	*Df(2R)Exel7142*	v		
*P{lacW}l(2)k07237^k07237^*	seq	*Df(2R)BSC133*	v	*P{lacW}k07237*	
*P{lacW}k07406*	seq	*Df(2R)ED3385*	v	*P{lacW}CG14480^k07406^*	
*P{lacW}l(2)k07509^k07509^*	seq		h	*P{lacW}k07509*	
*P{lacW}l(2)k07914^k07914^*	seq	*Df(2L)BSC340*, *Df(2L)BSC159*	v	*P{lacW}k07914*	
*P{lacW}l(2)k08002^k08002^*	seq	*18w^Delta7-35^*, *18w^k02701^*	v	*P{lacW}k08002*	
*P{lacW}l(2)k08407^k08407^*	seq	*Df(2R)BSC482*, *Df(2R)Exel7138*	v	*P{lacW}bdg^k08407^*	
*P{lacW}l(2)k08504^k08504^*	seq	*Df(2R)ED1735*, *Df(2R)Exel6055*	v	*P{lacW}ACC^k08504^*	*Nup44A^k08504^*
*P{lacW}l(2)k08816^k08816^*	seq	*Df(2R)BSC133*	v	*P{lacW}CG12744^k08816^*	
*P{lacW}l(2)k08915^k08915^*	seq	*Df(2L)BSC107*	v	*P{lacW}cbt^k08915^*	*ush^k08915^*
*P{lacW}l(2)k09202^k09202^*	seq	*Df(2R)Exel6066*	v, h	*P{lacW}k09202*	
*P{lacW}l(2)k09221^k09221^*	seq	*Df(2R)BSC132*	v	*P{lacW}k09221*	
*P{lacW}l(2)k09610^k09610^*	seq	*Df(2L)BSC107*	v	*P{lacW}ush^k09610^*	
*P{lacW}l(2)k09854^k09854^*	seq	*Df(2R)BSC433*	v	*P{lacW}GstS1^k09854^*	
*P{lacW}l(2)k09920^k09920^*	seq	*Df(2R)Exel6070*, *Df(2R)BSC430*	v	*P{lacW}k09920*	
*P{lacW}l(2)k09923^k09923^*	seq	*Df(2L)ED369*, *Df(2L)BSC185*	v	*P{lacW}k09923*	
*P{lacW}l(2)k09924^k09924^*	seq	*Df(2R)BSC347*	v	*P{lacW}k09924*	
*P{lacW}l(2)k10003a^k10003a^*	seq	*Df(2L)Exel8013*	v	*P{lacW}k10003a*	
*P{lacW}l(2)k10004^k10004^*	seq	*Df(2L)Exel9062*	v	*P{lacW}k10004*	
*P{lacW}l(2)k10113^k10113^*	seq	*Df(2L)ED479*	v	*P{lacW}k10113*	
*P{lacW}l(2)k10127^k10127^*	seq	*Df(2L)BSC172*	v	*P{lacW}Tfb5^k10127^*	*CG31917^k10127^*
*P{lacW}l(2)k10217^k10217^*	seq	*Df(2L)ED7853*	v	*P{lacW}CR45716^k10217^*	
*P{lacW}l(2)k10609^k10609^*	seq	*Df(2L)ED479*	v	*P{lacW}RapGAP1^k10609^*	
*P{lacW}l(2)k10815^k10815^*	seq	*Df(2R)BSC433*	v, h	*P{lacW}GstS1^k10815^*	
*P{lacW}l(2)k11120a^k11120a^*	seq	*Df(2R)BSC408*	v	*P{lacW}k11120a*	
*P{lacW}l(2)k11206^k11206^*	seq	*Df(2L)BSC109*	v	*P{lacW}pgant5^k11206^*	
*P{lacW}l(2)k11301^k11301^*	seq	*Df(2R)BSC433*	v	*P{lacW}GstS1^k11301^*	
*P{lacW}l(2)k11311^k11311^*	seq	*Df(2R)BSC347*	v	*P{lacW}k11311*	
*P{lacW}l(2)k11404^k11404^*	seq	*Df(2R)BSC463*	v	*P{lacW}CG34021^k11404^*	
*P{lacW}l(2)k11405^k11405^*	seq	*Df(2R)BSC433*	v	*P{lacW}GstS1^k11405^*	
*P{lacW}l(2)k13211^k13211^*	seq	*Df(2R)ED3952*	v	*P{lacW}k13211*	
*P{lacW}l(2)k13617^k13617^*	seq	*Df(2R)ED2751*	v	*P{lacW}Dek^k13617^*	
*P{lacW}l(2)k14206^k14206^*	seq	*Df(2L)BSC187*	v	*P{lacW}Tango1^k14206^*	
*P{lacW}l(2)k15617^k15617^*	seq	*Df(2R)ED2457*	v	*P{lacW}Dg^k15617^*	
*P{lacW}l(2)k16215^k16215^*	seq	*Df(2L)Exel7066*	v, h	*P{lacW}CG5953^k16215^*	
*P{lacW}l(2)k16406^k16406^*	seq	*Df(2L)BSC101*, *tsh^8^*	v	*P{lacW}k16406*	
*P{lacW}l(2)k16919^k16919^*	seq	*Df(2L)Exel7034*	v	*P{lacW}Bsg^k16919^*	
*P{PZ}l(2)03050^03050^*	seq	*Df(2R)Exel6071*	v	*P{PZ}ND-B14.7^03050^*	
*P{PZ}l(2)03497^03497^*	seq	*Df(2R)BSC280*, *Df(2R)BSC408*	v	*P{PZ}wun^03497^*	
*P{PZ}l(2)03605^03605^*	seq		h	*P{PZ}Tango1^103605^*	
*P{PZ}emm^1^*	seq	*Df(2R)BSC594*, *Df(2R)BSC883*	mf	*P{PZ}06268*	
*P{PZ}l(2)rL220^rL220^*	seq	*Df(2L)BSC142*	v	*P{PZ}cdc14^rL220^*	
*P{lacW}l(2)s2978^s2978^*	seq	*Df(2L)ED678*, *Df(2L)BSC203*	v	*P{lacW}rost^s2978^*	
*P{PZ}l(2)rK639^rK639^*	seq	*Df(2L)BSC290*	v	*P{PZ}rK639*	
*P{lacW}l(2)s1878^s1878^*	seq	*Df(2R)Exel7162*, *PCNA^2735^*, *PCNA^k00704^*, *PCNA^02448^*	ff	*P{lacW}PCNA^s1878^*	
*P{PZ}l(2)rAO135^rAO135^*	seq	*Df(2L)BSC102*, *tsh^8^*	v	*P{PZ}tsh^rAO135^*	
*P{lacW}l(2)k02107a^k02107a^*	*in situ*	*Df(2R)ED1742*, *Df(2R)BSC269*	v	*P{lacW}k02107a*	
*P{lacW}fs(2)k09833^k09833^*	*in situ*	*Df(2R)Exel7121*	ff	*P{lacW}k09833*	
*P{lacW}l(2)k07136^k07136^*	seq	*Df(2R)BSC698*	v	*P{lacW}k07136*	
*P{lacW}l(2)s4830^s4830^*	seq	*Df(2R)BSC661*	v	*P{lacW}CG43795^s4830^*	
*P{lacW}l(2)k00808^k00808^*	seq	*Df(2R)BSC603*	v	*P{lacW}mAChR-A^k00808^*	
*P{lacW}l(2)k03205^k03205^*	seq	*Df(2R)BSC607*	v	*P{lacW}NKAIN^k03205^*	*CR43466^k03205^*
*P{PZ}l(2)07806^07806^*	seq	*Df(2R)BSC814*	v	*P{PZ}CG34115^07806^*	
*P{PZ}l(2)07837^07837^*	seq	*Df(2R)BSC802*	v	*P{PZ}Fili^07837^*	
*P{PZ}l(2)10491^10491^*	seq	*Df(2R)BSC154*	v	*P{PZ}cnk^10491^*	
*P{PZ}l(2)10505^10505^*	seq	*Df(2R)ED3385*	v	*P{PZ}CG30105^10505^*	
*P{PZ}l(2)10333^10333^*	seq	*Df(2L)Exel6034*	v	*P{PZ}10333*	
*P{Mae-UAS.6.11}LA00508^LA00508^*	seq	*Df(2L)ED611*	v	*P{Mae-UAS.6.11}LA00508*	
*P{lacW}l(2)SH0108^SH0108^*	seq	*Df(2R)BSC274*	ff	*P{lacW}mip120^SH0108^*	
*P{lacW}bchs^SH0148^*	seq	*Df(2L)Exel7024*	v, h		
*P{lacW}l(2)SH0237^SH0237^*	seq	*Df(2R)BSC274*	v	*P{lacW}SH0237*	
*P{lacW}l(2)SH0294^SH0294^*	seq	*Df(2R)BSC597*	v	*P{lacW}wdp^SH0294^*	
*P{lacW}Phax^SH0641^*	seq		h		
*P{lacW}Galphas^SH0782^*	seq	*Df(2R)BSC601*	v		
*P{lacW}ItgaPS5^SH1114^*	seq	*Df(2R)BSC785*	v		
*P{lacW}CG4266^SH1128^*	seq	*Df(2R)BSC484*	v		
*P{lacW}l(2)SH1372^SH1372^*	seq	*Df(2L)ED343*	v, h	*P{lacW}Tsp26A^SH1372^*	
*P{lacW}l(2)SH1393^SH1393^*	seq		h	*P{lacW}Prosalpha7^SH1393^*	
*P{lacW}CG30015^SH1405^*	seq	*Df(2R)ED2098*, *Df(2R)BSC327*	v, h		
*P{lacW}mbm^SH1819^*	seq	*Df(2L)ED19*	v		
*P{lacW}Snp^SH1834^*	seq		h		
*P{lacW}l(2)SH1927^SH1927^*	seq	*Df(2R)BSC153*, *Df(2R)BSC259*	v	*P{lacW}SH1927*	
*P{lacW}hebe^SH2065^*	seq	*Df(2R)BSC131*	v, h		
*P{lacW}l(2)SH2138^SH2138^*	seq	*Df(2R)BSC770*	v	*P{lacW}SH2138*	
*P{lacW}oho48A^k06524^*	seq	*Df(2R)ED2247*, *Df(2R)ED2219*	v	*P{lacW}CG9005^k06524^*	

a *In situ*, insertion mapped by *in situ* hybridization to polytene chromosomes; seq, insertion mapped from sequence of insertion site.

b Phenotype in complementation tests: v, viable; ff, female fertile; mf, male fertile. An “h” indicates homozygotes were present in stocks (in some of these cases, complementation tests were not performed).

c When an insertion lies in the region of gene overlap, FlyBase uses the symbol of one gene in the P insertion symbol, and lists associated alleles for the other genes.

Table 2 shows the results of complementation tests with 18 stocks mapping recessive lethality to the P insertions. In these cases, strong arguments can be made for the lethality arising from disruption of the gene associated with the *P* element insertion based on noncomplementation with deletions and other mutations in the gene, or from the position of the P insertion relative to genic regions. For example, complementation tests with a deletion for the region of *dachsous* (*ds*) and a *ds* point mutation showed that the lethality of *P{PZ}l(2)01855^01855^* is attributable to *ds* disruption. This allowed the insertion symbol to be revised to *P{PZ}ds^01855^*.

**Table 2:** Insertions causing lethal phenotypes

Original Symbol	Noncomplementing Deletions and Mutations	Phenotype*^a^*	Complementing Deletions	New Insertion Symbol
*P{lacW}lola^k09901^*	*lola^00642^*, *lola^ORC4^*	l	*^b^**Df(3R)Exel6190*, *Df(3R)Exel6191*	
*P{lacW}l(2)k00705^k00705^*	*betaTub56D^YC0063^*, *Df(2R)BSC782*	l		*P{lacW}betaTub56D^k00705^*
*P{lacW}l(2)k05911^k05911^*	*CG31728^f02493^*, *Df(2L)BSC277*, *Df(2L)BSC768*	l		
*P{PZ}l(2)00248^00248^*	*Ef1alpha48D^01275^*, *Df(2R)BSC329*, *Df(2R)BSC879*	l		*P{lacW}EF1alpha48D^00248^*
*P{lacW}l(2)k09848^k09848^*	*CG7845^c00845^*, *Df(2R)ED1482*	l		
*P{PZ}l(2)01855^01855^*	*ds^UAO71^*, *Df(2L)Exel8003*	l		*P{PZ}ds^01855^*
*P{lacW}l(2)k13905^k13905^*	*Df(2L)glu-17C*	l		*P{lacW}Cyt-c-p^k13905^*
*P{lacW}l(2)k14805^k14805^*	*Adf1^01349^*, *Df(2R)ED1552*	l		*P{lacW}Adf1^k14805^*
*P{PZ}l(2)03563^03563^*	*Df(2R)BSC668*, *Df(2R)ED2354*	l		*P{PZ}Oaz^03563^*
*P{PZ}l(2)05287^05287^*	*CG12050^KG03759^*, *Df(2L)BSC302*, *Df(2L)Exel7080*	l		
*P{lacW}l(2)k10502^k10502^*	*Letm1^MB02246^*, *Df(2R)ED4061*	pl		*P{lacW}Letm1^k10502^*
*P{lacW}LeuRS^SH0501^*	*LeuRS^c03210^*, *Df(2L)drm-P2*	l		
*P{lacW}Ranbp11^SH0971^*	*Ranbp11^B217^*, *Df(2R)BSC398*	l		
*P{lacW}Arpc4^SH1036^*	*Arpc4^e00819^*, *Df(2L)Exel6015*	l		
*P{lacW}bsf^SH1181^*	*Df(2L)BSC149*, *Df(2L)Exel8038*	l		
*P{lacW}l(2)08770^k04808^*	*Ttd14^EY01823^*, *Ttd14^KG03769^*, *Df(2R)BSC335*	l/pl		*P{lacW}Ttd14^k04808^*
*P{PZ}l(2)08770^08770^*	*Ttd14^KG03769^*	l		*P{PZ}Ttd14^08770^*
*P{PZ}l(2)10685^10685^*	*^c^**Df(2L)Exel6004*, *Df(2L)ED108*	pl		

a Phenotype in complementation tests: l, lethal; pl, partially lethal; l/pl, lethal in combination with deletion and partially lethal in combination with other insertions.

b Results map lethality to *lola* in agreement with Bass *et al.* (2007), and not to region 94A as suggested by old sequence data.

c Unpublished results from the Gene Disruption Project showed failure to complement *P{lacW}l(2)10685^k00420^* (http://flybase.org/reports/FBal0008159.html).

Table 3 shows the results of complementation tests with 44 *P* element stocks that indicate lethality maps to the P insertion itself or to a closely linked site. Confidence that a P insertion is responsible for a phenotype and that the phenotype is not attributable to a hit-and-run mutation tightly linked to the P insertion increases as the size of the interval defined by noncomplementing deletions decreases. Although past experience has shown that noncomplementation with any deletion strongly predicts that phenotypes are caused by P insertions (Spradling *et al.* 1999), we cannot formally associate lethality with these insertions from our deletion crosses alone. For this reason, insertions within the transcribed regions of genes have been renamed to reflect their physical position, separate alleles have been named to represent the recessive lethality, and the respective FlyBase entries indicate (in the *Notes on Origin* section of allele reports, and the *Comments* section of insertion reports) that the insertion and the lethal locus may represent the same mutational event. For example, the insertion formerly called *P{lacW}l(2)k08601^k08601^* lies within the *Mef2* transcription unit. The insertion is now denoted by *P{lacW}Mef2^k08601^*, the coincident or closely linked recessive lethal mutation is denoted *l(2)k08601^k08601^*, and FlyBase entries indicate the two genetic elements may be the same entity. We made 28 such changes (Table 3, top section). No symbol updates were needed for nine other insertions within the transcribed portions of genes (Table 3, middle section). The seven insertions that lie outside the transcribed portions of genes (Table 3, bottom section) retain their original insertion names, because it is standard practice for FlyBase not to represent such insertions with separate symbols for insertions and lethal loci.

**Table 3:** Insertions with coincident or closely linked lethal mutations

Original Symbol	Noncomplementing Deletions and Mutations	Phenotype*^a^*	New Insertion Symbol	New Allele Symbols*^b^*	New Lethal Allele
Insertion and lethality may not be separable, but separate insertion and lethal locus named					
* P{lacW}l(2)SH0499^SH0499^*	*Df(2R)BSC334*, *Df(2R)Exel7153*	v-l	*P{lacW}MED9^SH0499^*	*CG42518^SH0499^*	*l(2)SH0499^SH0499^*
* P{lacW}l(2)45Ai^k00116^*	*Df(2R)BSC271*	l	*P{lacW}CG8078^k00116^*		*l(2)45Ai^k00116^*
* P{lacW}l(2)k02206^k02206^*	*Df(2R)BSC402*	l	*P{lacW}CG33785^k02206^*	*CG33786^k02206^*	*l(2)k02206^k02206^*
* P{lacW}l(2)k03201^k03201^*	*Df(2L)BSC296*, *Df(2L)BSC354*	l	*P{lacW}Sec61alpha^k03201^*		*l(2)k03201^k03201^*
* P{lacW}l(2)k04003k^04003^*	*Df(2L)ED623*	l	*P{lacW}Trs23^k04003^*		*l(2)k04003^k04003^*
* P{lacW}l(2)k04308^k04308^*	*Df(2R)BSC281*, *Df(2R)BSC303*	l	*P{lacW}gem^k04308^*		*l(2)k04308^k04308^*
* P{lacW}l(2)k05448^k05448^*	*Df(2L)BSC245*, *Df(2L)BSC290*, *Df(2L)Exel6034*	l	*P{lacW}CG5776^k05448^*		*l(2)k05448^k05448^*
* P{lacW}l(2)k06204^k06204^*	*Df(2R)BSC158*, *^c^**l(2)k06205^k06205^*	l	*P{lacW}Sec24AB^k06204^*	*CR45467^k06204^*	*l(2)k06204^k06204^*
* P{lacW}l(2)k06205^k06205^*	*Df(2R)BSC158*, *^c^**l(2)k06204^k06204^*	l	*P{lacW}Sec24AB^k06205^*	*CR45467^k06205^*	*l(2)k06204^k06205^*
* P{lacW}l(2)k06502^k06502^*	*Df(2L)ED270*	l	*P{lacW}CG11030^k06502^*		*l(2)k06502^k06502^*
* P{lacW}l(2)k07215^k07215^*	*Df(2L)BSC312*, *Df(2L)BSC302*, *Df(2L)Exel6047*	l	*P{lacW}CG9246^k07215^*		*l(2)k07215^k07215^*
* P{lacW}l(2)k07408^k07408^*	*Df(2R)BSC359*	l	*P{lacW}AsnRS-m^k07408^*		*l(2)k07408^k07408^*
* P{lacW}l(2)k08601^k08601^*	*Df(2R)X1*	l	*P{lacW}Mef2^k08601^*		*l(2)k08601^k08601^*
* P{lacW}l(2)k09328^k09328^*	*Df(2R)ED2308*	l	*P{lacW}CG17574^k09328^*		*l(2)k09328^k09328^*
* P{lacW}l(2)k10105^k10105^*	*Df(2L)BSC245*	l	*P{lacW}CG5287^k10105^*		*l(2)k10105^k10105^*
* P{lacW}l(2)k10239^k10239^*	*Df(2L)Exel7077*	l	*P{lacW}sick^k10239^*		*l(2)k10239^k10239^*
* P{lacW}l(2)k10317^k10317^*	*Df(2R)BSC360*	l	*P{lacW}MESK2^k10317^*		*l(2)k10317^k10317^*
* P{lacW}l(2)k12402^k12402^*	*Df(2R)BSC280*	l	*P{lacW}prel^k12402^*		*l(2)k12402^k12402^*
* P{lacW}l(2)k13412^k13412^*	*Df(2R)BSC279*	l	*P{lacW}CG8026^k13412^*	*CG45085^k13412^*	*l(2)k13412^k13412^*
* P{lacW}l(2)k13604^k13604^*	*Df(2L)ED21*	l	*P{lacW}CG3645^k13604^*		*l(2)k13604^k13604^*
* P{lacW}l(2)k16204^k16204^*	*Df(2R)Exel7164*	l	*P{lacW}HnRNP-K^k16204^*		*l(2)k16204^k16204^*
* P{lacW}l(2)k16805^k16805^*	*Df(2R)BSC668*	l	*P{lacW}Cpsf160^k16805^*		*l(2)k16805^k16805^*
* P{lacW}l(2)k17002^k17002^*	*Df(2R)X58-12*	l	*P{lacW}CG13510^k17002^*	*CG13511^k17002^*, *CG42565^k17002^*	*l(2)k17002^k17002^*
* P{PZ}l(2)04008^04008^*	*Df(2L)Exel6028*	l	*P{PZ}Ca-beta^04008^*		*l(2)04008^04008^*
* P{lacW}l(2)s4831^s4831^*	*Df(2R)Exel7164*	l	*P{lacW}HnRNP-K^s4831^*		*l(2)s4831^s4831^*
* P{PZ}l(2)rG270^rG270^*	*Df(2R)X58-12*	l	*P{PZ}Vps20^rG270^*		*l(2)rG270^rG270^*
* P{PZ}l(2)08492^08492^*	*Df(2R)Exel6054*, *Df(2R)Exel7092*	l	*P{PZ}CG30493^08492^*	*CG30496^08492^*	*l(2)08492^08492^*
* P{PZ}l(2)10481^10481^*	*Df(2R)Kr10*, *Df(2R)bw^VDe2L^Px^KR^*	pl	*P{PZ}lov^10481^*		*l(2)10481^10481^*
Insertion and lethality may not be separable, but separate insertion and lethal locus already existed.					
* P{lacW}CG30007^SH0071^*	*Df(2R)BSC158*	l			
* P{lacW}CG8414^SH0180^*	*Df(2R)ED2457*	l			
* P{lacW}ZnT49B^SH0360^*	*Df(2R)BSC485*, *Df(2R)Exel7121*	l			
* P{lacW}CG6094^SH0578^*	*Df(2L)BSC210*, *Df(2L)Exel7048*	l			
* P{lacW}CG9641^SH1104^*	*Df(2L)ED206*	l			
* P{lacW}x16^SH1297^*	*Df(2L)BSC108*, *Df(2L)ED6569*	l			
* P{lacW}IntS8^SH1314^*	*Df(2R)BSC382*	l			
* P{lacW}clumsy^SH1386^*	*Df(2L)Exel6047*	l			
* P{lacW}YL-1^SH1685^*	*Df(2L)BSC145*	l			
Insertion not within the transcribed portion of a gene. Insertion and lethality may not be separable, but no separate lethal locus named.					
* P{lacW}l(2)k00302^k00302^*	*Df(2L)BSC892*	l			
* P{lacW}l(2)k07803^k07803^*	*Df(2R)BSC298*, *Df(2R)B5*	l			
* P{lacW}l(2)k09035^k09035^*	*Df(2L)Exel6034*	l			
* P{lacW}l(2)k11328^k11328^*	*Df(2L)BSC277*, *Df(2L)BSC892*, *Df(2L)BSC290*, *Df(2L)ED784*	l			
* P{lacW}l(2)k15817^k15817^*	*Df(2L)BSC241*	l			
* P{lacW}l(2)37Db^k16106^*	*Df(2L)Exel6042*	l			
* P{PZ}l(2)03996^03996^*	*Df(2R)BSC268*	l			

a Phenotype in complementation tests: l, lethal; pl, partially lethal; v-l, homozygous viable but lethal in combination with deletions.

b When an insertion lies in the region of gene overlap, FlyBase uses the symbol of one gene in the P insertion symbol and lists associated alleles for the other genes.

c Original allele symbol.

Table 4 shows the results of complementation tests that allowed us to refine the mapping of 21 P insertions that had previously been mapped to polytene chromosome bands by *in situ* hybridization. Because we have no sequence localizations for these insertions, we cannot assign symbols reflecting their proximity to annotated genes. Consequently, they retain their original symbols indicating they are members of genetically defined, recessive lethal, or sterile complementation groups.

**Table 4:** Insertions with refined mapping

Original Symbol	Noncomplementing Deletions	Phenotype*^a^*	Complementing Deletions	Refined Mapping
*P{PZ}ms(2)43C^00919^*	*Df(2R)ED1715*, *Df(2R)ED1673*	ms		43A4–D3, 2R:7326951–7533553
*P{A92}disd^1^*	*Df(2R)BSC152*, *Df(2R)BSC298*	l		46C1–7, 2R:9875312–9958120
*P{A92}hall^1^*	*Df(2R)BSC334*, *Df(2R)ED3610*	l		55B2–C4, 2R:18051197–18357037
*P{lacW}l(2)35Fg^k08106a^*	*Df(2L)ED1102*	l	*Df(2L)Exel7066*	36A1–36A10; 2L:16457328–16684883
*P{SUPor-P}l(2)55Db^425-1^*	*Df(2R)BSC335*	l		55C6–F1, 2R:18398361–18755764
*P{PZ}l(2)37Ad^02660b^*	*Df(2L)Exel6042*, *Df(2L)OD15*, *Df(2L)ED1202*	l		37B8–10, 2L:18973942–19049265
*P{lacW}l(2)43Bd^k13522^*	*Df(2R)BSC263*, *Df(2R)ED1715*	l	*Df(2R)BSC264*	43A4–B2, 2R:7326951–7395885
*P{hsneo}fs(2)neo4^1^*	*Df(2L)ED5878*	l/fs	*Df(2L)BSC106*, *Df(2L)ED19*, *Df(2L)ED50001*	21B1–3, 2L:72671–159063
*P{hsneo}fs(2)neo5^1^*	*Df(2L)BSC150*, *Df(2L)Exel6049*	l		40A5–D3, 2L:21828252–22019106
*P{lacW}oho55DE^k13104^*	*Df(2R)BSC399*	l	*Df(2R)Exel7157*	55D1–E2, 2R:18503870–18621522
*P{lacW}l(2)10685^k00420^*	*Df(2L)Exel6004*	l		21E4–F1, 2L:1074079–1158137
*P{BS2.7B}l(2)32BCa^13^*	*Df(2L)BSC213*	l	*Df(2L)BSC241*	32B1–C1, 2L:10809118–11001451
*P{lacW}l(2)08492^k10320^*	*Df(2R)Exel6054*, *Df(2R)Exel7092*	l		43E9–12, 2R:7665795–7708707
*P{lacW}l(2)05287^k16804b^*	*Df(2L)BSC302*, *Df(2L)Exel7080*	l		39A1–2, 2L:21070044–21102742
*P{ry11}fs(2)ry6^1^*	*Df(2L)BSC107*	l/fs		21C2–E2, 2L:431096–574741
*P{ry11}fs(2)ry11^1^*	*Df(2L)BSC107*	l/fs		21C2–E2, 2L:431096–574741
*P{PZ}l(2)02836a^02836a^*	*Df(2R)Exel6063*	l		52F6–53C4, 2R:16187888–16386515
*P{PZ}l(2)03832a^03832a^*	*Df(2L)ED1466*	l	*^b^**Df(2L)BSC103*	39E7–40A5, 2L:21676769–21828548
*P{PZ}l(2)04535b^04535b^*	*Df(2R)BSC326*	l		42A14–C7, 2R:6236062–6746030
*P{PZ}l(2)43Bb^04614a^*	*Df(2R)BSC263*	l	*Df(2R)BSC264*, *Df(2R)ED1715*	42F2–43A4, 2R:7146864–7326951
*P{lacW}l(2)k12502b^k12502b^*	*Df(2R)Kr10*	l		60F2–5, 2R:25061964–25288936

a Phenotype in complementation tests: l, lethal; l/fs, lethal with female sterile escapers; ms, male sterile.

b Figure 2 in Han *et al.* (2003) suggests insertion lies to the right of *Df(2L)BSC103*.

In summary, we have clarified the basis for phenotypic effects seen in 178 *P* element insertion stocks in the Bloomington Stock Center collection. This has allowed us to give the stocks less ambiguous genotypes, and to revise several potentially misleading FlyBase entries. We expect this information to be valuable to *Drosophila* geneticists using these insertions in experiments examining the effects of disrupting specific gene regions.

## Supplementary Material

HTML Page - index.htslp
