# Supplementary material for: Phenotypes Associated with Second Chromosome P Element Insertions in Drosophila melanogaster
Source: G3 (Bethesda). 2016 Jun 10;6(8):2665–70. doi: 10.1534/g3.116.030940 (PMC4978919; doi:10.1534/g3.116.030940)
Supplement: HTML Page - index.htslp [file supp_6_8_2665__index.html]

Phenotypes Associated with Second Chromosome P Element Insertions in Drosophila melanogaster — HTML Page - index.htslp 

# Phenotypes Associated with Second Chromosome *P* Element Insertions in *Drosophila melanogaster*

## Supplemental Material for Kahsai, Millburn, and Cook, 2016

**Files in this Data Supplement:**

- Table S1 - Control crosses. (.xlsx, 22 KB)
